# Enhancement of hypothalamic-pituitary activity in male athletes: evidence of a novel hormonal mechanism of physical conditioning

**DOI:** 10.1186/s12902-019-0443-7

**Published:** 2019-11-01

**Authors:** Flavio A. Cadegiani, Claudio E. Kater

**Affiliations:** Division of Endocrinology and Metabolism, Department of Medicine, Escola Paulista de Medicina, Universidade Federal de São Paulo, Rua Pedro de Toledo 781 – 13th floor, São Paulo, SP 04039-032 Brazil

**Keywords:** Hormonal conditioning, Anterior pituitary, Hypothalamus, Sports endocrinology

## Abstract

**Background:**

Exercise is known to induce multiple beneficial conditioning processes. Conversely, although exercise may generate several hormonal effects, an intrinsic hormonal conditioning process has not been reported. In the Endocrine and Metabolic Responses on Overtraining Syndrome (EROS) study, we observed inherent and independent conditioning processes of the hypothalamic-pituitary axes in athletes. Our objective is to describe the theory of the novel hormonal conditioning mechanism using the findings from the EROS study.

**Methods:**

In this cross-sectional study, we selected 25 healthy athletes (ATL) and 12 non-physically active healthy controls (NPAC), 18–50 years old, males, with BMI 20–30 kg/m^2^, with similar baseline characteristics, who underwent gold-standard exercise-independent tests: cosyntropin stimulation test (CST) and insulin tolerance test (ITT), to evaluate cortisol response to CST, and ACTH, cortisol, GH, and prolactin responses to an ITT.

**Results:**

Responses to ITT were significantly earlier and higher in ATL than NPAC for cortisol [Mean ± SD: 21.7 ± 3.1 vs 16.9 ± 4.1 μg/dL; *p* < 0.001], GH [Median (95% CI): 12.73 (1.1–38.1) vs 4.80 (0.33–27.36) μg/L; *p* = 0.015], and prolactin [24.3 (10.5–67.45) vs 10.50 (6.21–43.44) ng/mL; *p* = 0.002]. Cortisol response to CST was similar between ATL and NPAC. During ITT, cortisol, GH, and ACTH mean increase in ATL were 52.2, 265.2, and 18.6% higher than NPAC, respectively. Prolactin response was absent in NPAC, while present in ATL.

**Conclusions:**

We found sufficient evidence to propose the existence of a diffuse enhancement of the hypothalamic-pituitary activity in athletes, not restricted to any axis, showing an intrinsic and independent process of “hormonal conditioning” in athletes, similar to those observed in the cardiovascular and neuromuscular systems. This novel conditioning process may be the missing link for understanding the improved responses observed in athletes to harmful situations, traumas, infections, inflammations, and psychiatric conditions.

## Background

Exercise, including leisure-time activities, has been shown to decrease overall mortality [1], whereas physical inactivity may independently justify almost 10% of deaths [2]. The decreased mortality associated with exercises is explained by the improvement of multiple aspects of health, including primary [3] and secondary [4] prevention of cardiovascular events. It has also been linked to unexpected outcomes, such as reduction of the incidence, recurrence and mortality [5] of some types of cancer. Moreover, physical activity also leads to improvement of all domains of life quality [6] and reduction of the incidence of psychiatric [7] and neurodegenerative disorders [8].

However, the underlying mechanisms that lead to the benefits from exercising, as well as the improvement of performance, are not yet fully elucidated. Many of the elicited benefits resulted from multiple conditioning processes that occur in response to repeated and progressive athletic training. These include adaptations of the cardiovascular and autonomic systems [9, 10], improvements in skeletal muscle [11], and pain modulation [2], as well as neurological [12] and psychiatric [13] responses.

**Fig. 1:**
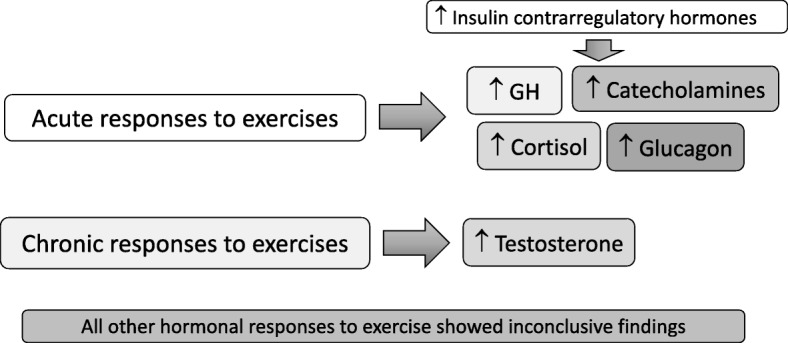
Current knowledge on hormonal responses to exercises

Although exercise has been shown to induce acute and chronic hormonal effects [14–16] (Fig. 1), an inherent hormonal conditioning process has not been reported to date. While basal and resting hormones have been compared between healthy athletes (ATL) and non-physically active control subjects (NPAC) in previous studies [17], none have assessed hormone responses to any type of exercise-independent stimulus. Moreover, although hormonal responses have been determined in ATL and compared to athletes affected by overtraining syndrome (OTS) [17–19], it remains unclear whether the responses in ATL were different from those in NPAC. Also, most of the previous hormonal responses to tests performed in ATL were stimulated by exercise. Such exercise-induced tests depend on musculoskeletal and cardiovascular signaling to the hypothalamus and pituitary and therefore any differences in hormonal responses could be attributed to differences in signaling, and not specifically to the hormonal changes.

Since the adaptive changes as presently known do not fully explain the benefits and progressive performance associated to exercise, further pathways must be explored.

The Endocrine and Metabolic Responses on Overtraining Syndrome (EROS) study was initially designed to evaluate hormone and metabolic responses of OTS-affected athletes, by comparison with ATL and NPAC, using gold-standard and exercise-independent tests [20, 21]. We used cosyntropin stimulation tests (CST) for direct evaluation of the adrenocortical reserve, and insulin tolerance tests (ITT) to evaluate the integrity of the hypothalamus-pituitary (corticotrophic, somatotrophic, and lactotrophic) axes. Thusfar, both arms of the EROS study that evaluated the hormone responsiveness to stimulation to stimulation, regardless of physical exertions, showed an optimized response in healthy athletes by up to 3–4 times, compared to healthy non-athletes. Although the primary objective was not to uncover physiological adaptations in healthy athletes, the interesting finding that the exacerbated hormonal response to demands occurred in distinct hypothalamic-pituitary axes allowed us hypothesize that there is a diffuse, inherent, and exercise-independent conditioning process of at least three hypothalamus-pituitary axes, in response to athletic training. This mechanism may explain some not previously understood findings in athetes, and predict new benefits from exercising. In this article, we explore the characteristics of the novel mechanism of hormonal conditioning response to exercise to provide, by performing a comprehensive joint analysis using the findings from two of the arms of the EROS study.

## Methods

### Subjects

A detailed description of the design, methods, subject selection criteria, and baseline characteristics for the EROS study is available in a depository (https://osf.io/bhpq9/). The study was approved by the ethical committee of the Federal University of São Paulo (approval number: 1093965). For the analysis in the present article, we included two groups: ATL and NPAC. Selected results from both the EROS-HPA axis [20] and the EROS-STRESS [21] arms of the EROS study were included in this analysis.

We recruited subjects through social media (Facebook and Instagram); the aim of the recruitment process was to include ATL and NPAC who were eligible for the study. A preliminary analysis of the candidates was done by e-mail correspondence, and included questions regarding age, sex, and approximate body weight and height. Based on the candidate’s responses, their approximate body mass index (BMI) was calculated. If no exclusion criteria were identified, an individual interview was then scheduled.

At the interview, body weight and height were verified using high precision weight and height scales. Questions regarding other conditions, use of medications or hormones, and characteristics of the sport (in the case of athletes) were also asked, and age was confirmed by verification of an identity card. All subjects had to fulfill the following inclusion criteria at this point: male; aged between 18 and 50 years; BMI between 20.0 and 32.9 kg/m^2^; absence of previous psychiatric disorders and use of centrally-acting drugs; and absence of any hormonal therapy in the previous 6 months.

The following additional inclusion criteria regarding training aspects were required from all ATL regarding training level: exercise at least four times a week, for a total of > 300 min a week, with moderate-to-vigorous training intensity (self-perception comparing own training with that of others, based on the Talk Test), and continuous training for the current sport(s) for at least 6 months, without interruption for > 30 days. The quantification of training load was recorded by the coach of each athlete, but not in a systematic way, as the recruitment occurred transversally in order to collect real-life training data (i.e.*,* it was not controlled). We required a minimum amount of physical activity for potential exercise-induced adaptions.

NPAC were required to: (1) fulfill the initial inclusion criteria, (2) be sedentary (without any physical activity, including light exercises) for at least 3 years, and (3) no history of exercise that would fulfill the criteria for ATL.

Candidates who fulfilled criteria were selected. After signing a written consent, the remaining subjects underwent biochemical examination to exclude confounding disorders and prevent inclusion of subjects with altered basal and stimulated hormone levels due to inflammation, infection, kidney disease, lipid metabolism abnormalities, vitamin deficiencies, or obvious hormonal dysfunctions. In ATL, exams were performed from 36 to 48 h after the last training session. The biochemical inclusion criteria used is listed in Table 1.

**Table 1:** Biochemical inclusion criteria for the EROS study

	Range required for inclusion	Assay method
Ultrasensitive C-reactive protein (CRP)	<3 mg/dL	Latex-intensified immunoturbidimetry
Erythrocyte sedimentation rate (ESR)	<25 mm/h	Automated spontaneous sedimentation method
Creatinine (and TFG)	<1.5 mg/dL (> 60 mL/min)	Jaffe enzymatic assay
Hematocrit	36–54%	Automated assay
Neutrophils	1000–9000 /mm^3^	Automated assay
Creatine kinase (CK)	< 5000 U/L	Calorimetric activity assay; International Federation of Clinical Chemistry
Alanine aminotransferase (ALT)	<50 U/L	Calorimetric activity assays
Aspartate aminotransferase (AST)	<50 U/L	Calorimetric activity assays
Ferritin	20–1000 ng/dL	Chemiluminescence assay
Vitamin B12	>180 pg/mL	Chemiluminescence assay
Fasting glucose	<100 mg/dL	Enzymatic assay of hexokinase
Total testosterone	>200 ng/dL	Chemiluminescence assay
TSH	<5 μIU/mL	Chemiluminescence assay

*TGF* Calculated estimated glomerular filtration rate, *TSH* Thyroid stimulating hormone

### Design

After the selection process, the subjects underwent basal biochemical tests, hormonal responses to stimulation tests, and evaluation of sleep, psychological, social and eating patterns, and analysis of the body metabolism and composition, as part of the different arms of the EROS study [20–23], with a maximum interval from the beginning to the end of the tests of 10 days. All tests and the collection of blood for analysis were medically supervised. Blood or plasma collection tubes were checked before and after each collection to ensure that an appropriate tube type was used for the biomarker and that each subject was properly identified. Following collection, the tubes were immediately centrifuged or analyzed to prevent loss of quality of the collected material. For the present study, we analyzed the hormonal responses to stimulation tests.

We evaluated the peripheral component of the hypothalamic-pituitary-adrenal (HPA) axis with a CST, which directly evaluates adrenal responses to synthetic adrenocorticotropic hormone (ACTH) [24], and whose impaired responses are indicative of primary adrenocortical dysfunction. Then we evaluated the central component of the HPA axis with an ITT, which evaluates the integrity of the hypothalamus and pituitary [24], and whose normal response requires absence of dysfunctions in all levels of the HPA axis (hypothalamus, pituitary, and adrenals). Whenever adrenal responses are normal to the CST, any abnormality observed in an ITT must be located in the hypothalamus or in the pituitary [24]. We also evaluated growth hormone (GH) and prolactin responses to the ITT, as well as glucose changes and clinical behavior during a hypoglycemic episode. These tests were part of the “EROS-HPA axis” [20] and “EROS-Stress” [21] arms of the EROS study.

### Methodology

For the CST, subjects were required to fast for 8 h and to have had the last training session at least 72 h (3 days) prior to the test, and to arrive at the laboratory at 7:30 h in the morning. They sat in blood-drawing chairs and rested for 30 min. Ten mL of blood (divided into two EDTA tubes) was collected before and 30 and 60 min after an intravenous administration of 250 μg of cosyntropin (as recommended by the guidelines of Endocrinology societies [24]) for analysis of cortisol.

Subjects underwent the ITT 48 h after the CST, following an 8-h fasting and a minimum of 120 h without exercising. Subjects arrived at the laboratory at 7:30 h in the morning, were seated in a blood-drawing chair, and rested for 30 min. Then, 0.1 IU/kg of regular insulin was administered intravenously after blood collection (10 mL in ethylenediaminetetraacetic acid (EDTA) and plasma tubes) at time zero (baseline). Capillary glucose was checked every 5 min from time 10 min after insulin administration, or whenever subjects reported symptoms. Blood for time one was collected when: 1) capillary glucose was < 30 mg/dL without symptoms; 2) subjects classified symptoms of hypoglycemia as moderate to severe (5–10) regarding either adrenergic (shakiness, cold sweating, heart palpitations, or pallor) or neuroglycopenic (sleepiness, mood changes, or unrest) symptoms, or both; or 3) if capillary glucose was < 45 mg/dL in presence of any symptom. If after 40 min none of these three criteria was achieved, an extra 0.05 IU/kg of regular insulin was administered intravenously; and again after an additional 40 min, if none of the criteria was achieved. Finally, if hypoglycemia did not occur, the subject would be withdrawn from the study due to likely insulin resistance (which makes it unfeasible to perform a proper ITT test). However, none of the patients required a third dose of insulin.

After time one blood collection, 10 mL of 50% glucose solution was infused intravenously and high-glycemic index and pure carbohydrate food (lemon or strawberry dairy-free sorbet, Diletto, Brazil) was offered ad libitum. Ten mL of blood was collected again, 30 min after the hypoglycemic episode (time two). In all blood samples we determined cortisol, ACTH, GH, prolactin and glucose, as well as the absolute ACTH/cortisol ratio at all times during ITT [24–26]. During the ITT we evaluated the time-to-hypoglycemia (min) since insulin administration and self-reported intensity of adrenergic and neuroglycopenic symptoms on a scale of 0 to 10 (0 = asymptomatic, 10 = severe symptoms). We did not perform the 60 min for all athletes, as the first round (a “pilot” evaluation, with three sedentary and three healthy athletes) did not disclose differences for any of the hormones between 30 and 60 min after hypoglycemia. Also, protocols for ITT admit variations regarding time for the blood collection, and whether the time for blood collect depended the hypoglycemic episode or not.

Due to the risk of severe hypoglycemia during ITT, subcutaneous glucagon pens were always available (GlucaGen HypoKit, 1 μg, NovoNordisk), as well as 20 mL-syringes containing 50% glucose solution and an automated external defibrillator (AED).

Basal and hypoglycemia-induced serum cortisol, plasma ACTH, serum GH and serum prolactin levels were determined by commercially available electrochemiluminescence assays, that were previously validated, standardized and tested. The detection limits for ACTH and GH were 5.0 pg/mL and 0.05 μg/L, respectively, but there was no minimal analytical limit for the other markers. The intra- and inter-assay coefficients of variability of all the biochemical markers measured in all arms of the EROS study were below 3.0 and 3.5%, respectively.

We evaluated basal and hypoglycemia-induced and absolute changes in the levels of cortisol, ACTH and prolactin during the ITT (from time zero to time two); GH changes were not determined because of its wide pulse amplitude. Mean time-to-hypoglycemia and intensity of adrenergic and neuroglycopenic symptoms were also compared between groups.

In addition, a 7-day dietary record with specific calorie and macronutrient account, and self-reported sleeping patterns, social and psychological characteristics, basal muscular, inflammatory, immunologic and hormonal parameters, and body composition and metabolism were evaluated in all selected participants.

### Statistical analysis

Nonparametric ANOVA (Kruskal-Wallis) was performed whenever criteria for normality were not met, and *post-hoc* adjusted Dunn’s test was performed for subgroup analyses whenever *p* < 0.05. Conversely, one-way ANOVA was used when criteria for normality were met, followed by Dunnett’s T3 and Tukey *post-hoc* analysis for subgroup analyses. All statistical tests were performed using IBM-SPSS statistics version 24.0 software (IBM, USA). Raw data is available at https://osf.io/bhpq9/.

### Post-hoc joint analysis

We joined the responses to CST and ITT, obtained from the EROS-HPA axis and EROS-STRESS arms of the EROS, including analysis.

We did not compare the magnitude of the responses between GH, prolactin, cortisol, and ACTH, as hormones have distinct behaviors with respect to the physiological amplitude of responses to stimulations. Instead, we compared differences between ATL and NPAC responsiveness within each hormone, through the ratios between hormonal responses in athletes and non-athletes.

We analyzed the context of the findings, from which we proposed the new hypothesis of hormonal conditioning process that athletes undergo.

## Results

### Subject selection and baseline characteristics

Initially, 59 participants were enrolled, including 46 ATL and 13 NPAC. In the first interview, 17 subjects were excluded: 12 were currently using or had used hormones in the last 6 months; four used centrally-acting drugs; three due to insufficient calorie intake, one did not fill the BMI criteria; and one healthy subject did not meet ATL criteria. One ATL was excluded for low testosterone and one NPAC due to a diagnosis of type 2 diabetes mellitus. Finally, three ATL withdrew during the study due to personal reasons. The average BMI (ATL = 26.7 kg/m^2^ and NPAC = 25.9 kg/m^2^, age (ATL = 32.7 years and NPAC = 33.2 years) were similar between ATL and NPAC, while all participants had no differences in terms of presence or absence of clinical conditions of baseline biochemical data that could lead to differences in hormonal responses.

### Cortisol response to CST

Cortisol responses to CST were similar at all times between ATL and NPAC, as shown in the EROS-HPA axis arm of the present study (30).

### Cortisol response to ITT

Basal cortisol levels were similar between ATL and NPAC; however, they were significantly higher in ATL during (*p* = 0.016) and 30 min after hypoglycemia (*p* < 0.0001), as well as the mean cortisol increase in response to ITT (*p* = 0.026) (Table 2).

**Table 2:** Basal and stimulated cortisol levels in response to an insulin tolerance test (ITT) in athletes (ATL) and non-physically active control subjects (NPAC)

Hormone	ATL (*n* = 25)	NPAC (*n* = 12)	*P*-value (ATL × NPAC)
Cortisol (μg/dL) (mean ± SD)			
Basal	12.5 ± 3.1	10.9 ± 2.8	*ns*
During hypoglycemia	15.9 ± 5.3	11.8 ± 3.1	0.016
P-value *of* ***Δ*** *cortisol (x basal)*	0.018	*NS*	
30 Minutes after hypoglycemia	21.7 ± 3.1	16.9 ± 4.1	<0.0001
P-value *of* ***Δ*** *cortisol (x basal)*	<0.0001	0.0004	

Abbreviations: *SD* standard deviation, *ns* non significant

A significant early cortisol rise (during hypoglycemia) was present only in the ATL group (*p* = 0.018, compared to basal levels), whereas late responses were observed for both groups (ATL < 0.0001; NPAC = 0.0004, compared to basal levels).

### ACTH response to ITT

A consistent and significant rise in ACTH levels at both times (during and 30 min after hypoglycemia) was observed only in the ATL group (*p* = 0.0001, compared to basal levels), whereas in NPAC only the late rise was significant (*p* = 0.001) (Table 3).

**Table 3:** Basal and stimulated ACTH levels in response to an insulin tolerance test (ITT) in athletes (ATL) and non-physically active control subjects (NPAC)

Hormone	ATL (*n* = 25)	NPAC (*n* = 12)	*P*-value (ATL × NPAC)
ACTH (pg/mL) (median; 95% CI)			
Basal	18.7 (6.5–37.8)	21.4 (8.7–37.8)	*ns*
During hypoglycemia	57.8 (7.3–229.5)	29.5 (14.8–191.7)	*ns*
P-value *of* ***Δ*** *ACTH (x basal)*	0.0001	*NS*	
30 Minutes after hypoglycemia	59.9 (22.1–195.7)	51.4 (22.7–137.5)	*ns*
P-value *of* ***Δ*** *ACTH (x basal)*	<0.0001	0.001	

Abbreviations: *ACTH* adrenocorticotropic hormone, *CI* confidence interval, *ns* non significant

Although the significant early response of ACTH to ITT was observed only in the ATL group, and the median ACTH level in NPAC was as lower as almost twice than in ATL, the difference between groups was not statistically significant.

### GH response to ITT

GH levels were consistently and significantly higher in ATL than NPAC at all times (*p* = 0.003, *p* = 0.006, and *p* = 0.015, for basal, early, and late response, respectively) (Table 4).

**Table 4:** Basal and stimulated GH levels in response to an insulin tolerance test (ITT) in athletes (ATL) and non-physically active control subjects (NPAC)

Hormone	ATL (*n* = 25)	NPAC (*n* = 12)	*P*-value (ATL × NPAC)
GH (μg/L) (median; 95% CI)			
Basal	0.26 (0.1–1.26)	0.06 (0.03–0.47)	0.003
During hypoglycemia	2.50 (0.08–40.94)	0.16 (0.05–8.13)	0.006
*P*-value *of* ***Δ*** *GH (x basal)*	0.0002	NS	
30 Minutes after hypoglycemia	12.73 (1.1–38.1)	4.80 (0.33–27.36)	0.015
*P*-value *of* ***Δ*** *GH (x basal)*	<0.0001	0.0002	

Abbreviations: *CI* confidence interval, *GH* growth hormone, *ns* non significant

Within group comparisons show that a significant early GH response (during hypoglycemia) was only observed in ATL (*p* = 0.0002, versus basal levels), whereas late responses were observed in both ATL (*p* < 0.0001) and NPAC (p = 0.0002).

Basal GH levels were undetectable (< 0.05 μg/L) in one ATL (4%) and five NPAC (42%), and remained undetectable during hypoglycemia in three NPAC (25%) but in none ATL.

### Prolactin response to ITT

Basal prolactin levels were similar between groups, but responses during and 30 min after hypoglycemia were significantly higher in ATL than in NPAC (*p* = 0.01 and *p* = 0.002, respectively), as well as prolactin change during ITT (p = 0.01) (Table 5).

**Table 5:** Basal and stimulated prolactin levels in response to an insulin tolerance test (ITT) in athletes (ATL) and non-physically active control subjects (NPAC)

Hormone	ATL (*n* = 25)	NPAC (*n* = 12)	*P*-value (ATL × NPAC)
Prolactin (ng/mL) (median; 95% CI)			
Basal	12.1 (7.2–23.0)	10.6 (7.9–15.7)	*NS*
During hypoglycemia	17.8 (10.0–63.4)	12.2 (7.2–15.9)	0.01
*P*-value ***Δ*** *Prolactin (x basal)*	0.0032	NS	
30 Minutes after hypoglycemia	24.3 (10.5–67.4)	10.5 (6.2–43.4)	0.002
*P*-value ***Δ*** *Prolactin (x basal)*	<0.0001	n/s (0.98)	–

Abbreviation: *CI* confidence interval, *ns* non significant

Prolactin significantly increased in ATL at both early (*p* = 0.0032) and late (*p* < 0.0001) responses; in NPAC, there were no increases to ITT, but instead a slight and non-significant paradoxical reduction.

### Glucose behavior on ITT

Both basal glucose (ATL = 81.4 ± 5.1 mg/dL; NPAC = 84.8 ± 6.4 mg/dL) and glucose during hypoglycemia (ATL = 18.0 ± 8.3 mg/dL; NPAC = 26.2 ± 12.5 mg/dL) were similar between ATL and NPAC.

### Differences of the responsiveness to ITT between healthy athletes and non-athletes

During ITT, cortisol mean increase was 9.2 ± 3.7 in ATL versus 6.0 ± 3.9 in NPAC, respectively, depicting a significant difference of 53.3% between the magnitude of the cortisol responses to ITT (*p* = 0.026).

Median ACTH increase was 45.1 (22.1–195.7) in ATL versus 38.0 (0.5–108.8) in NPAC, depicting a difference of 18.6% between the magnitude of the ACTH responses to ITT, although not significant.

Median GH increase was 12.73 (1.1–38.1) in ATL versus 4.80 (0.33–27.36) in NPAC, depicting a difference of 265.2% between the magnitude of the GH responses to ITT, which although highly variable, was still significant (*p* = 0.015).

With respect to prolactin, a significant increase only occurred in ATL [13.1 (− 5.3 − + 54.5)], while a slight but paradoxical decrease was observed in NPAC [1.2 (− 4.8 − + 30.5)], as mentioned above. The lack of prolactin responses to ITT in NPAC precludes an analysis of the ratio between the magnitude of prolactin responses.

**Fig. 2:**
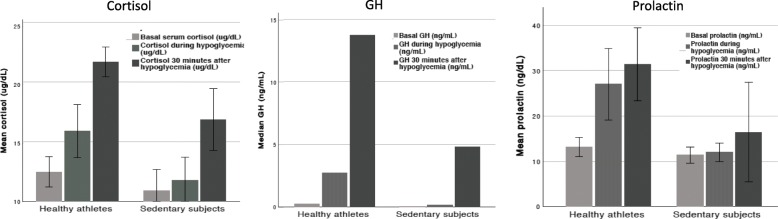
Cortisol, GH, and prolactin responses to an Insulin Tolerance Test (ITT)

Table 6 depicts the differences between the level of hormonal responsiveness to ITT between ATL and NPAC. Figure 2 illustrates the differences between cortisol, GH, and prolactin responses to ITT. ACTH response was not illustrated as it did not depict significant differences between ATL and NPAC. All differences remained significant after adjustments for muscle and fat mass, although these were not included in the present manuscript.

**Table 6:** Differences of the magnitude of the hormonal responses between healthy athletes and non athletes

Hormone	ATL (*n* = 25)	NPAC (*n* = 12)	Difference between the responses of ATL and NPAC (*P*-value)
Cortisol change during ITT (μg/dL)	9.2 ± 3.7	6.0 ± 3.9	+ 53.3% (*p* = 0.026)
*P*-value (*overall* increase)	<0.0001	0.001	–
ACTH change during ITT (pg/mL)	45.1 (22.1–195.7)	38.0 (0.5–108.8)	+ 18.6% (*p* = *ns*)
*P*-value *(overall increase)*	<0.0001	0.0065	–
GH change during ITT (μg/L)	12.57 (1.01–37.7)	4.74 (0.3–27.28)	+ 265.2% (*p* = 0.016)
*P*-value *(overall increase)*	<0.0001	0.0004	–
Prolactin change during ITT (ng/mL)	+ 13.1 (−5.3 − + 54.5)	−1.2 (−4.8 − + 30.5)	*n/a* (only ATL responded) (*p* = 0.01)
*P*-value *(overall increase)*	0.0001	n/s (0.88)	–

ACTH, GH, and prolactin: median and 95% CI; Cortisol: mean ± SD

Abbreviation: *CI* confidence interval, *SD* standard deviation, *ns* non significant

## Discussion

### Conditioning processes from exercising

Conditioning processes in cardiovascular, autonomic, musculoskeletal, psychiatric, and neurologic systems resulting from physical activity play an important role for further performance improvement among athletes. Additionally, beneficial effects go beyond exercise capacity, as they may improve multiple metabolic pathways, chronic pain, mood states, reflexes, and cardiovascular outcomes [1–12]. However, current findings on adaptations to exercise do not fully explain the multiple advantages related to physical activity [14–16].

Despite some favorable hormonal effects of chronic athletic training [14–16], further hormonal improvements not necessarily related to physical exertion have not been demonstrated in athletes.

### Remarkable findings of the EROS study

In the present study, direct adrenal stimulation did not disclose differences between ATL and NPAC, which demonstrate that the *fasciculata* layer of the adrenal glands does not present any intrinsic conditioning effect in response to stimulation. However, the overall response to an ITT caused a prominent cortocotrophic, somatotrophic and possibly lactotrophic [20, 21] response in ATL, compared to NPAC.

Early ITT responses were only observed in ATL, whereas differences in GH, prolactin, and cortisol levels were even more remarkable 30 min after hypoglycemia. Importantly, despite a possible insulin hypersensitivity usually observed in athletes, which could lead to differences in glucose levels and responsiveness to the ITT, glucose levels were similar between groups initially and during hypoglycemia, and all participants achieved glucose levels to induce insulin contra-regulatory stimulation, reinforcing the equality of ITT conditions and excluding differences due to the intensity of hypoglycemia.

### Specific findings of hormone responses

Aside from the enhanced hormonal responses, we observed that prolactin showed an exclusive response in athletes, not found in non-physically active, which may explain the lack of large descriptions of the lactotrophic responses to stimulation tests in the literature. However, the role of the prolactin responses in athletes is unknown.

ACTH and GH are pulsatile hormones, leading to responses of great amplitude, and explains the behavior of their responses in the present study. In addition, ACTH is a hormone whose half-life is very short, which hinders its precise evaluation in stimulation tests, as stated by Endocrinology Societies [27], and is likely the reason why ACTH did not depict differences between ATL and NPAC.

The exacerbated response observed in the HPA axis of the athletes was not found when adrenals were directly stimulated through the CST, showing that the conditioning process of the HPA axis does not occur in the adrenal glands, but centrally instead.

These findings were not related to differences in sleeping or eating patterns, as groups had similar sleeping quality, duration, and hygiene, and tests were conducted after a period of fasting, respectively.

In regards with differences as being secondary to muscle of fat mass, there is no sufficient literature to support their influences, except for the negative influence of body fat on GH release. Despite the lack of supporting literature for these influences, we employed overestimated adjustments for muscle and fat mass, and differences between healthy athletes and sedentary remained significant.

### Evidence of the enhancement of hypothalamic-pituitary activity in athletes as a novel conditioning process

The ubiquitous optimization of hormonal responses and an exclusive prolactin response to demands observed in athletes, independent from any other characteristic, strengthen the hypothesis of the presence of a diffuse enhancement of the hypothalamic-pituitary activity, not restricted to any axis, as an additional intrinsic conditioning adaptive process that athletes undergo.

Considering that:

- Different from other tests, the ITT directly tests the integrity of the hypothalamic-pituitary axes, without any interference from other systems, including cardiovascular and musculoskeletal;
- Unlike previous studies that employed exercise-dependent tests, in which differences in responses could be attributed to differences in performance, the ITT does not have any influence from the physical capacity or performance, which evens athletes and non-athletes;
- Differences between athletes and sedentary are not justified by differences in age, sex, or BMI, since groups had similar baseline characteristics, while differences in hormonal responses remained after further adjustments;
- A large number of clinical and biochemical confounding factors, including the presence of clinical conditions, biochemical abnormalities, and influences from eating and sleeping patterns, were excluded during the selection process;
- There are not any potential biases from what the existing literature has already demonstrated regarding the tests performed;
- The criteria and hormonal functional tests employed in the present study are highly standardized, and fully satisfy the strict endocrinological criteria for an appropriate test;
- Results were highly distinct, with important differences in the mean or median, and narrow standard deviations and confidence intervals, respectively, and almost absence of overlapping results; and
- There seems to be no plausible explanation other than the existence of a “hyperresponsiveness” of the hypothalamic-pituitary axes to justify the present findings in athletes,

we found sufficient supportive data to propose the hypothesis of the occurrence of intrinsic and independent “hormonal conditioning” process in athletes, located centrally in the hypothalamus-pituitary axis, similar to those observed in the cardiovascular system and muscle tissue. We also inferred that the enhancement of the hypothalamic-pituitary axes occurred in a non-selective manner, as the optimization of the responses were indistinguishable between these axes.

**Fig. 3:**
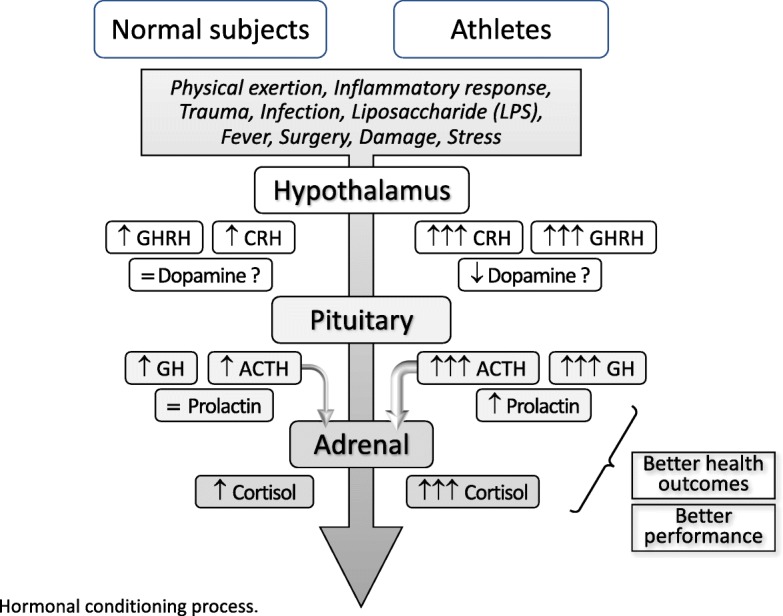
Hormonal conditioning process

**Fig. 4:**
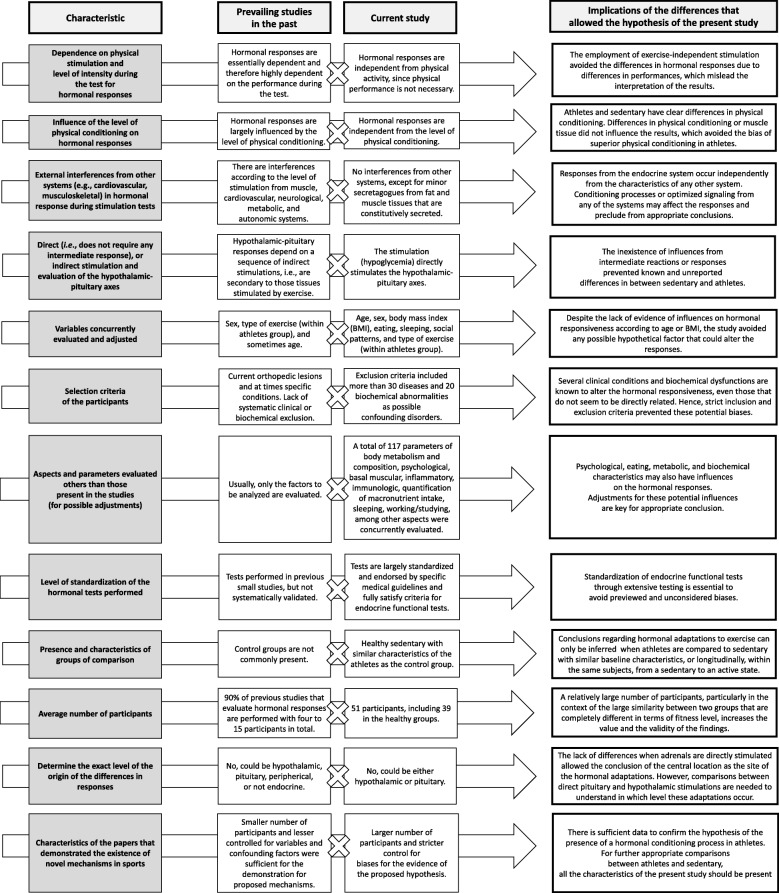
Differences of the characteristics between the current and the past studies, and how these differences implied in the evidence of the existence of an inherent, independent and diffuse hypothalamic-pituitary hormonal conditioning process in athletes

However, whether this adaptive process occurs at the tertiary (hypothalamus) or secondary (pituitary) level is unknown, since the stimulation test used in the present study does not distinguish between them. The “hormonal conditioning” process is illustrated in Fig. 3. The highlights of the differences between the previous data and the current findings, and how these differences provided the evidence for the hypothesis of the existence of the “hormonal conditioning” process, are shown in Fig. 4.

Finally, besides the findings of the optimized hormonal responses to a stimulation test, additional hormonal adaptations were observed in the EROS study, including increased testosterone and dopamine levels [27], although this was not the aim of the present manuscript.

### Implications of the enhanced hypothalamic-pituitary activity in athletes

The current study may fill both gaps in our knowledge of hormone adaptation to exercise: first, by showing that hormonal responses in ATL are enhanced in terms of speed and amplitude, and second, by demonstrating that this optimization goes beyond exercise, as the ITT is an exercise-independent test. If only exercise-dependent stimulation tests were used, it would not be possible to verify whether these changes extend to other types of stimulation. This is because exercise-based tests may induce a secondary hormonal increase due to enhancement in neurological, sympathetic, autonomic, and cardiovascular signaling. Thus, we would be unable to conclude whether any exacerbation of hormonal responses would occur if these other signaling pathways were not present.

Regardless of the test employed, we have demonstrated that exacerbation of hormone responses to stimulation play a key role in improving physical performance, as faster overall hormone response may account for better initiation of a training load (which is particularly important in short and intense or anaerobic sports), while wider amplitude response may improve the pace during a training session (which may be helpful in long-distance endurance sports).

Additional demonstration of the improvement of hormone responses to other types of stimuli may help explain the benefits of physical activity that go beyond exercise, including better responses to acute infections and unexpected harmful situations, improved metabolic responses, an enhanced sense of well-being, and increased life expectancy [1, 2], at least from moderate-to-intense exercises, or > 6 METs (metabolic equivalent of task), as employed in the EROS study.

### Limitations

The present study was performed with healthy athletes that practiced concurrent strength and endurance exercises. Whether the enhancement of hypothalamic-pituitary activity occurs in females, or athletes of exclusive endurance, strength, or explosion sports is unknown. Moreover, the initial objective of the EROS study was to evaluate hormonal, clinical, and metabolic behaviors of overtraining syndrome, by comparing with two control groups: of healthy athletes and healthy non-athletes. The serendipitous findings of healthy athletes, when compared to non-athletes, allowed the hypothesis presented in this study.

## Conclusions

We found evidence of the presence of a diffuse enhancement of the hypothalamic-pituitary activity in healthy athletes, not restricted to any axis. Therefore, we demonstrated sufficient data to propose the hypothesis of the occurrence of an intrinsic and independent “hormonal conditioning” process that athletes undergo, similar to those observed in the cardiovascular system and muscle tissue. This novel conditioning process unveiled by our study may be the missing link for understanding the underlying mechanisms of improvement observed in several responses observed in athletes to harmful situations, traumas, infections, inflammations, and psychiatric conditions. Nevertheless, further longitudinal studies are necessary to confirm our hypothesis.

## Data Availability

The raw data is fully available at https://osf.io/bhpq9/.

## Abbreviations

ACTH: adrenocorticotropic hormone
AED: automated external defibrillator
ATL: healthy athletes
BMI: body mass index
CST: cosyntropin stimulation test
EDTA: ethylenediaminetetraacetic acid
EROS: Endocrine and Metabolic Responses on Overtraining Syndrome
GH: growth hormone
HPA: hypothalamic-pituitary-adrenal
ITT: insulin tolerance test
MET: metabolic equivalent of task
NPAC: non-physically active control subjects
OTS: overtraining syndrome
